# Cutaneous Myiasis in Rural Haiti

**DOI:** 10.31486/toj.19.0073

**Published:** 2020

**Authors:** Amanda Theppote, Yvens Laborde, Leise Knoepp, Shontell Thomas, Obinna N. Nnedu

**Affiliations:** ^1^ Department of Internal Medicine, Ochsner Clinic Foundation, New Orleans, LA; ^2^ The University of Queensland Faculty of Medicine, Ochsner Clinical School, New Orleans, LA; ^3^ Medical Director, Global Health Education, Medical Director, Public Health, Ochsner Clinic Foundation, New Orleans, LA; ^4^ Department of Obstetrics and Gynecology, Ochsner Clinic Foundation, New Orleans, LA; ^5^ Department of Infectious Diseases, Ochsner Clinic Foundation, New Orleans, LA

**Keywords:** *Myiasis*, *screw worm infection*

## Abstract

**Background:** Myiasis is a disease caused by the infestation of human tissue by the larval stage of various flies. It has been identified in sub-Saharan Africa and in tropical parts of the Americas. Cases have also been identified among travelers returning to the United States. Infestations may involve any part of the body, including the scalp, and open wounds may become infected with these larvae. The primary cause of wound myiasis in the western hemisphere is *Cochliomyia hominivorax*.

**Case Report:** We present a case of wound myiasis in an adult Haitian male with a persistent wound for 2 years. To our knowledge, only 1 other report of wound myiasis in Haiti caused by *C hominivorax* has been published.

**Conclusion:** Wound myiasis can occur in many tropical regions of the world, including Haiti. Because of the prevalence of global travel, clinicians should be familiar with the condition's diagnosis and management.

## INTRODUCTION

Myiasis is the infestation of vertebrate animals and humans with dipterous larvae that feed on the host's living or dead tissue.^1^ The method of invasion into tissue differs, depending on the species of fly.^2,3^ Cutaneous myiasis may manifest clinically in 3 ways: furuncular, migratory, or wound type. Furuncular myiasis is the most common presentation and occurs when 1 or more larvae infest the skin and cause a furuncle.^3^ Migratory or creeping dermal myiasis typically starts with a papule that progresses to a pruritic, serpentine lesion as the larvae burrow through the skin.^4^ Wound myiasis occurs when fly larvae are directly inoculated into or in the vicinity of a wound. To our knowledge, only 1 other report has been published of myiasis caused by *Cochliomyia hominivorax* in 2 patients in Haiti.^5^ We describe another case of myiasis in a Haitian male whose preexisting skin lesion was secondarily infested with *C hominivorax* larvae.

## CASE REPORT

A 26-year-old male presented to a rural clinic in northern Haiti with a 2-year history of a nodular wound on the back of his head. He denied trauma or memory of an insect bite to the area. He was unsure how the wound started. The wound was initially painless, but the patient noted that the nodule had slowly grown larger and had become painful. He denied constitutional symptoms and relevant medical history. His human immunodeficiency virus status was unknown; testing was not available at the clinic at the time of his presentation. He worked outdoors on a farm and had not sought medical care prior to presenting to the clinic.

Initial examination revealed a 3 × 2 × 1-cm nodule in the occipital area of the head. Purulent fluid was draining from the sinus tracts within the nodule. The examination was also notable for cervical and postauricular lymphadenopathy.

In clinic, the nodule was irrigated with sterile saline and chlorhexidine. The sinus tracts were opened, and the nodule was debrided. Following debridement, hemostasis was achieved, and the patient's wound was dressed. He was started on trimethoprim-sulfamethoxazole (TMP-SMX) 160-800 mg and discharged with instructions to follow up the next day for dressing change. On the following day, the patient's wound still exuded a fair amount of drainage, so it was reopened and again debrided, the wound was dressed, and amoxicillin-clavulanic acid 875-125 mg was added to the patient's antibiotic regimen.

**Figure 1.**
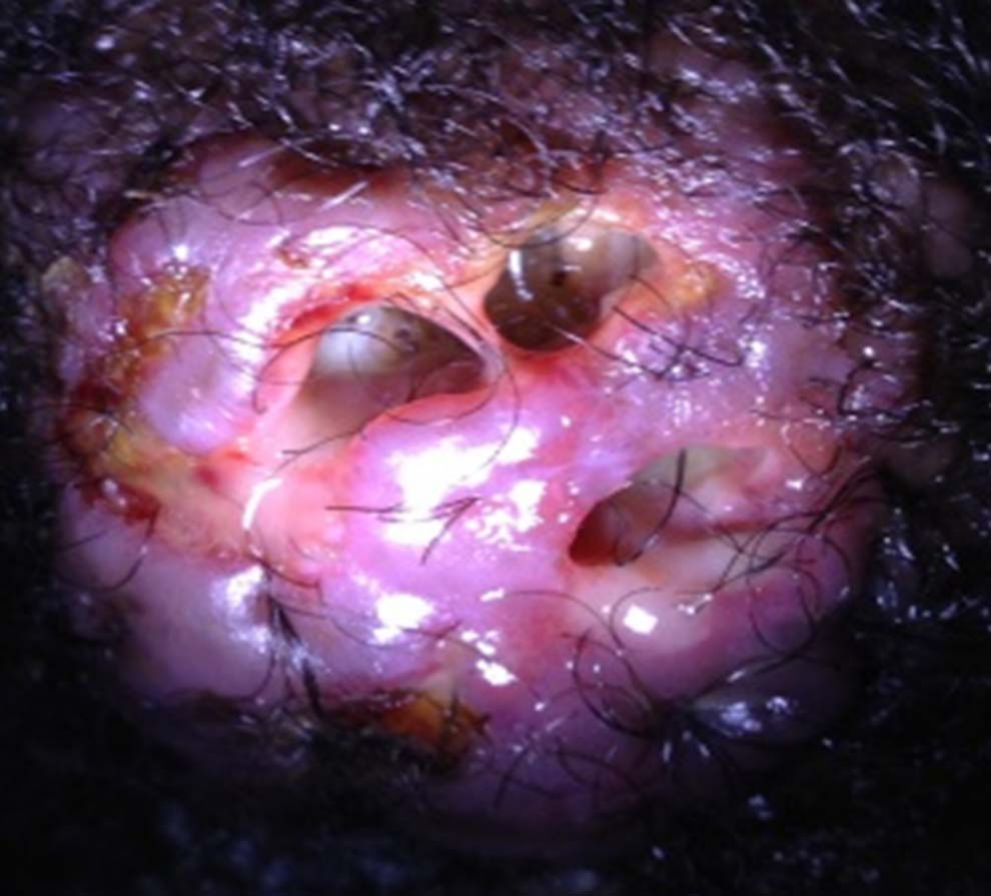
**Multiple larvae were present within the patient's wound.**

**Figure 2.**
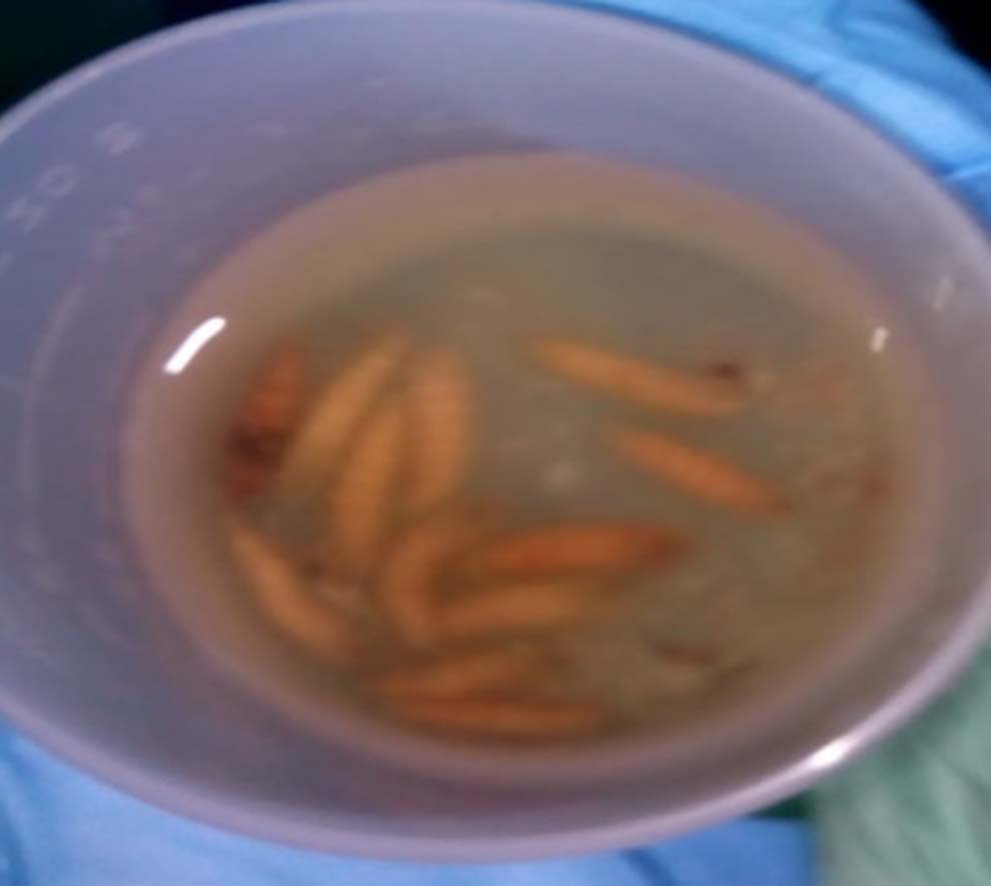
**Twelve larvae were extracted from the patient's wound.**

The patient presented to the clinic on the third day with complaints of feeling a crawling sensation at the back of his head. When the dressings were removed, larvae were visualized within the tracts of the wound (Figure 1). With the patient in the prone position on the examination table, the sinus tracts were expanded with forceps. The larvae were visualized with the assistance of a flashlight and extracted with tweezers. In total, 12 larvae were removed (Figure 2). His wound was cleansed with chlorhexidine and dressed. He was instructed to complete an additional 10-day course of TMP-SMX and amoxicillin-clavulanic acid. He followed up approximately 1 year later to the clinic, and the wound had healed completely.

## DISCUSSION

Wound myiasis is commonly caused by *C hominivorax* in South and Central America, including Haiti.^1,5^ *C hominivorax*, also known as the New World screwworm (NWS), is an obligatory wound parasite that lays eggs in or on the edges of wounds. The female deposits multiple eggs at once.^1,2^ After a short incubation period of nearly 24 hours, the eggs hatch and feed upon live tissue for 4 to 8 days.^1^ During the maturation process, the larvae burrow or screw deep into tissue and create the deep pocket-like lesions characteristic of NWS that cause significant local pain.^2,6^ *C hominivorax* can be identified by the dark pigmentation on their dorsal tracheal trunks.^6^ Predisposing factors for wound myiasis include poor hygiene, poor social conditions, and open wounds.^1^ International travel has become a risk factor as well. The number of tourists who crossed international boundaries increased from 537 million in 1995 to 1.135 billion in 2014.^7^ Myiasis is 1 of the 5 most common travel-related skin infections, accounting for 3.5% to 9% of all travel-related skin infections.^8,9^

There are 3 primary ways to manage myiasis. The first treatment modality is to expose the larvae to toxic substances. Topical or oral ivermectin is toxic to the larvae and may lead to death. The drawback to this approach is that the death of larvae within tissues may provoke a brisk localized inflammatory response. The second treatment modality is to create localized hypoxia by using petroleum jelly or paraffin to cover the sinus tracts. The larvae emerge from the sinus tracts for oxygen, allowing for ease of removal. The third and most common way to manage these wounds is mechanical or surgical removal of the larvae.

## CONCLUSION

Myiasis is a tropical disease associated with poor living conditions in developing countries and may also be seen in international travelers. Knowledge of the diagnosis and management of myiasis is not only important for clinicians working in endemic areas but also for those working in nonendemic areas because of widespread international travel.

## Notes

This article meets the Accreditation Council for Graduate Medical Education and the American Board of Medical Specialties Maintenance of Certification competencies for Patient Care and Medical Knowledge.
